# Drug resistance mechanisms in *Mycobacterium tuberculosis i*nfection and challenges in vaccine development

**DOI:** 10.3389/fphar.2026.1762214

**Published:** 2026-02-09

**Authors:** Shuying Zhang, Juan Cheng, Yuanyuan Tang

**Affiliations:** 1 Department of Laboratory Medicine, Weifang No. 2 People’s Hospital, Weifang, China; 2 Translational Medical Center, Weifang No. 2 People’s Hospital, Weifang, China

**Keywords:** drug resistance mechanisms, immune evasion, immunoinformatic, *Mycobacterium tuberculosis*, vaccine development

## Abstract

Drug-resistant *Mycobacterium tuberculosis*(Mtb) has become a global public health crisis, and its diverse drug resistance jointly reduces the effectiveness of antibacterial drugs. Mtb resistance is not merely genetic but involves a synergistic interplay of cell wall remodeling, metabolic reprogramming, and epigenetic regulation, all of which are closely linked to its capacity for immune evasion. These mechanisms lead to the failure of traditional treatments, exacerbating the prolongation of treatment duration, the increase in mortality rate and the spread of drug-resistant bacteria. Vaccine research has gradually become a key strategy for preventing and controlling the spread of drug-resistant tuberculosis. This review synthesizes these multifaceted resistance pathways and parallels them with the challenges in vaccine development, highlighting the limited efficacy of Bacillus Calmette-Guérin and the promise of next-generation candidates. It further explores the landscape of novel therapeutic strategies, including new drugs like bedaquiline and host-directed therapies. In the future, efforts should be focused on the development of multivalent vaccines, the integration of chemoimmunotherapy, and the sharing of global monitoring data to contribute to the ultimate goal of eliminating tuberculosis.

## 1 Introduction

Tuberculosis (TB), caused by *Mycobacterium tuberculosis* (Mtb), remains one of the most formidable infectious diseases globally. TB still poses a significant public health challenge due to its high mortality and transmissibility. Despite significant advances in medical science, TB continues to claim approximately 1.6 million people annually and affects millions more worldwide (Laws et al., 2022). The ability to infect a substantial fraction of the global population, with an estimated one-third harboring latent infection, underscores the complexity of disease control and eradication efforts (Carabalí-Isajar et al., 2023). The disease predominantly targets the lungs but can also manifest extrapulmonary involvement (Jellal et al., 2025). Given TB remains a critical focus for public health interventions, research, and policy-making due to its global burden and the socioeconomic impact (Turbawaty et al., 2025).

One of the most important challenges in TB control is the emergence and dissemination of drug-resistant strains of Mtb. The emergence of multidrug-resistant (MDR) and extensively drug-resistant (XDR) TB strains have significantly compromised the efficacy of conventional antibiotic regimens, leading to increased treatment failures and higher mortality rates (Wang et al., 2024). The development of resistance arises from complex mechanisms, including genetic mutations conferring resistance to first- and second-line anti-TB drugs, as well as phenotypic tolerance facilitated by host-pathogen interactions (Li et al., 2022; Mishra et al., 2021). Meanwhile, Vaccination remains a cornerstone for TB prevention, yet the current *Bacillus* Calmette-Guérin (BCG) vaccine exhibits variable and often limited efficacy against adult pulmonary TB, which is the most common and transmissible form (Schrager et al., 2020; Lange et al., 2022). On the other hand, BCG confers robust protection against severe childhood TB manifestations, its effectiveness wanes with age and does not reliably prevent pulmonary disease in adolescents and adults (Mart et al., 2022). In fact, the variability in BCG efficacy is attributed to multiple factors, including genetic differences among vaccine strains, host immune response heterogeneity, and environmental influences (Asadian et al., 2022; Yang et al., 2021). Furthermore, BCG vaccination is contraindicated in the immunocompromised individuals with HIV, limiting its utility in high-risk populations (Larson et al., 2023). The intersection of drug resistance and immunocompromise highlights the need for integrated approaches. These limitations further emphasize the urgent need for improved vaccines that induce durable and protective immunity, particularly targeting pulmonary TB.

In light of these challenges, current researches need to focus on deciphering the molecular basis of drug resistance and the difference of host-pathogen interactions to guide novel interventions. Recent studies have illustrated various resistance pathways, including mutations affecting drug targets and efflux pump mechanisms, as well as the ability to manipulate host immune responses to evade clearance (Bobba et al., 2023; Ilesanmi et al., 2024). Additionally, immunological study continued to clarify the roles of specific T-cell subsets, cytokines, and macrophage states in determining infection outcomes (Fang et al., 2025). These insights have spurred interest in host-directed therapies, which aim to modulate immune responses to enhance bacterial clearance and reduce tissue damage (Fang et al., 2025; Bobba and Khader, 2023). Concurrently, vaccine research has expanded to include novel platforms such as recombinant viral vectors, multi-antigenic formulations, and mucosal delivery strategies to elicit robust pulmonary immunity (Kilinç et al., 2021; Dijkman et al., 2021). However, several challenges remain in translating mechanistic knowledge into effective clinical interventions. The complex heterogeneity of Mtb strains, varying host genetic backgrounds, and the pathogen’s sophisticated immune evasion strategies complicate vaccine design and therapeutic efficacy (Guler et al., 2021). Moreover, the emergence of drug-resistant strains demands new drugs with novel targets and mechanisms, as well as adjunctive therapies to overcome resistance and enhance treatment outcomes (Kotliarova et al., 2024). The limited protective efficacy of BCG also emphasized the necessity for innovative vaccine candidates and delivery methods (Xie et al., 2025). Additionally, the integration of computational tools, such as immunoinformatics and *in silico* modeling, offers promising ways to accelerate vaccine and drug development pipelines (Chakraborty et al., 2024; Russo et al., 2020).

This review aims to synthesize current knowledge on the mechanisms of Mtb drug resistance and the challenges encountered in TB vaccine development and potential new drug development. We analyze the molecular underpinnings of resistance, the interplay between Mtb and host immunity, and the implications for therapeutic and prophylactic strategies. Furthermore, we discuss the limitations of existing vaccines and emerging approaches that seek to enhance protective efficacy and address the global TB burden. In this review, we focus on how emerging epigenetic insights can bridge molecular mechanisms of drug resistance and host immune regulation with translational opportunities in TB diagnostics and vaccine development, while critically evaluating the challenges of clinical implementation.

## 2 Drug resistance mechanisms of Mtb

**FIGURE 1:**
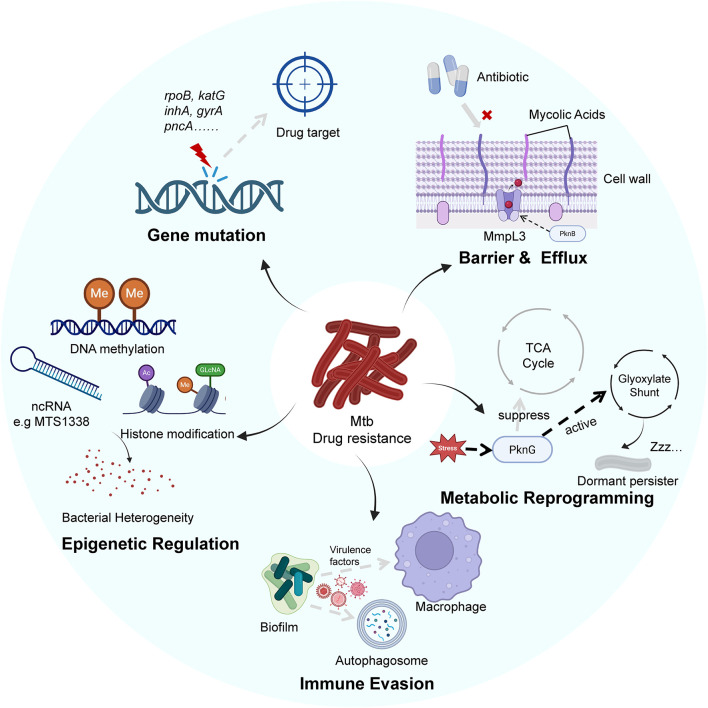
The description of multifaceted drug resistance mechanisms in Mtb. The bacterium resides within a host macrophage, where it inhibits phagosome maturation and secretes virulence factors, establishing a protective intracellular niche. The complex and multi-layered cell wall, maintained by transporters like MmpL3, acts as a primary barrier to antibiotic influx. Intracellularly, genetic mutations alter drug targets, rendering antibiotics ineffective. Under stress, Mtb undergoes metabolic reprogramming to enter a drug-tolerant non-replicating persister state. Concurrently, epigenetic mechanisms generate bacterial heterogeneity and promote adaptive resistance.

The persistent global burden of TB is exacerbated by the remarkable adaptability of Mtb, which employs multifaceted strategies to survive within the host and resist antibiotic therapy. A comprehensive understanding of TB pathogenesis and treatment failure requires an integrated examination of several interconnected domains. This includes the dynamic interplay between host immune responses and bacterial evasion, the genetic mutations that confer classical drug resistance, the epigenetic regulation in bacterial persistence and adaptive changes in bacterial cell wall architecture and core metabolism. These complicated mechanisms of drug resistance in Mtb are collectively illustrated in Figure 1.

### 2.1 Characteristics of host immune responses and immune evasion mechanisms

Host immune responses to Mtb infection are primarily mediated by cellular immunity, involving both innate and adaptive components. Macrophages act as the first line of defense by recognizing Mtb through pattern recognition receptors such as Toll-like receptors (TLRs), leading to phagocytosis and the production of pro-inflammatory cytokines and chemokines (Wei et al., 2024). Granuloma formation, which consists of aggregates of macrophages, T cells, and neutrophils, is a hallmark of TB pathology and helps contain the pathogen (Vu et al., 2024). CD4^+^ T cells, especially Th1 subsets, play a central role by secreting interferon-gamma (IFN-γ) to activate macrophages, while CD8^+^ T cells contribute through cytotoxic activity and cytokine production (Liu et al., 2025). Natural killer (NK) cells and other innate lymphoid cells also participate in early immune responses (Zhang H. et al., 2025). The balance between pro-inflammatory and anti-inflammatory responses is critical to avoid tissue damage or bacterial persistence (Källenius et al., 2024).

Mtb has evolved sophisticated immune evasion mechanisms to subvert host immunity. It modulates antigen presentation, inhibits phagosome maturation, and interferes with host cell death pathways (Pal et al., 2022). Secretory proteins like ESAT-6 and PE/PPE family members manipulate host signaling pathways to dampen immune responses and promote bacterial survival (Passos et al., 2024). Mtb also exploits host ubiquitination and autophagy machinery for immune evasion (Shariq et al., 2023). Additionally, Mtb suppresses macrophage activity by altering cytokine profiles and inhibiting the production of reactive oxygen and nitrogen species, such as nitric oxide (NO), which are essential for bacterial killing (Kong et al., 2025). Besides, Mtb also regulates cell death pathways, including pyroptosis, to balance immune activation and bacterial survival (Abrahams and Besra, 2021).

Drug-resistant Mtb strains exhibit distinct interactions with the host immune system. Resistance mechanisms such as gene duplications may alter antigen expression and immune recognition (Espinoza et al., 2025). Enhanced biofilm formation in resistant strains provides a protective niche against both antibiotics and immune effectors, contributing to phenotypic tolerance and immune evasion (Sarangi et al., 2024). The interplay between immune evasion and drug resistance complicates vaccine development. Vaccine candidates targeting conserved antigens involved in immune evasion such as EsxG and EsxH, are being explored to enhance host responses (Martinez-O et al., 2023). Host-directed therapies have shown promise in preclinical studies. These include modulators like everolimus, which influence autophagy and inflammation, as well as immunotherapeutic agents like withaferin A, which promote macrophage polarization and T cell memory (Raien et al., 2023). Strategies to boost host defenses such as increasing glutathione synthesis in macrophages, are also investigated as adjuncts for therapy (Nasiri et al., 2024).

Additionally, MDR and XDR Mtb strains pose significant treatment challenges. These strains interact with the host immune system in ways distinct from drug-sensitive strains, often inducing altered immune responses that contribute to their persistence and pathogenicity. For example, infections with isoniazid-resistant Mtb preferentially activate the cGAS-STING/STAT1 pathway in macrophages, driving overexpression of interferon-stimulated genes and indicating a unique immunometabolic reprogramming (Savijoki et al., 2021). Concurrently, patients with drug-resistant TB exhibit a hyperinflammatory state marked by elevated plasma levels of cytokines, which correlates with increased disease severity (Sampath et al., 2023). The relationship between immune evasion and drug resistance is bidirectional and mutually reinforcing. Mtb utilizes proteins like Rv2652c to suppress proinflammatory responses via the NF-κB pathway, thereby establishing a protective niche that supports bacterial survival and the expansion of drug-resistant populations (Li and Dou, 2024). Similarly, the bacterial protein MoxR1 inhibits host cell death pathways and disrupts cellular bioenergetics through metabolic reprogramming, collectively weakening host defenses and promoting phenotypic antibiotic tolerance (Quadir et al., 2023). Persistent Mtb populations are further shielded within biofilms or mesenchymal stem cells, which provide sanctuaries from immune effectors and antibiotics, linking dormancy to drug resistance (Devi et al., 2023). At the molecular level, resistance mechanisms such as gene duplications in mmpL5 and embC-A may also alter antigenic profiles and modulate host–pathogen interactions (Espinoza et al., 2025). Bacterial sensors like MmpL10 enable Mtb to adapt to host immune signals such as interferon-γ and enhance both survival and drug tolerance (Ahmed et al., 2022). Thus, immune evasion strategies facilitate the emergence and maintenance of drug-resistant strains, while resistance mechanisms in turn influence immune recognition.

In summary, the characteristics of host immune responses, centered on cellular immunity, and Mtb’s immune evasion mechanisms are closely linked. Immune dysregulation due to evasion strategies or host factors facilitates disease progression and drug resistance.

### 2.2 Gene mutations and drug target alterations

The development of drug resistance in Mtb is predominantly driven by genetic mutations that alter drug targets or drug activation pathways, reducing the efficacy of anti-tubercular agents. For first-line drugs, isoniazid (INH) resistance is primarily associated with mutations in the katG gene, which encodes the catalase-peroxidase enzyme responsible for activating INH (Rivière et al., 2020). The Ser315Thr substitution in katG is the most frequent and clinically significant mutation and it severely compromises INH activation (Krittanan et al., 2022; Sultana et al., 2024). Additionally, mutations in the promoter region of the inhA gene such as C15T, lead to overexpression of the InhA, resulting in target-based resistance even when INH is activated (Zhang et al., 2023; The CRyPTIC Consortium, 2022). Traditional molecular diagnostics often focus on detecting katG S315 and inhA promoter mutations for INH resistance profiling (Dou et al., 2025). In contrast, Rifampicin (RIF) resistance is a key marker for MDR-TB, with over 95% of cases linked to mutations in the rpoB gene encoding the β-subunit of RNA polymerase (Farhat et al., 2024). The S450L mutation is predominant worldwide, accounting for 37%–54% of RIF-resistant isolates, and induces conformational changes that reduce RIF binding affinity (Zeng et al., 2021). Similarly, Pyrazinamide (PZA) resistance mainly arises from mutations in the pncA gene, which encodes pyrazinamidase. These mutations, including common substitutions at codons 12 and 85, impair enzyme activity and prevent conversion of PZA to its active form, pyrazinoic acid (Mahmood et al., 2022). Although mutations in rpsA and panD have been reported in PZA resistance, their contributions are considered less significant compared to pncA and require further validation (Liu et al., 2018).

Resistance to second-line injectable drugs is commonly associated with mutations in the rrs gene (Sharma et al., 2023). Fluoroquinolone (FQ) resistance, a component of extensively drug-resistant tuberculosis (XDR-TB), is primarily driven by mutations in the gyrase genes. The gyrA D94G substitution is a key marker of high-level FQ resistance, as these mutations reduce drug binding affinity (Bi et al., 2021). Bedaquiline (BDQ) resistance primarily involves mutations in the mmpR5 (Rv0678) gene, which encodes a transcriptional repressor. The mmpR5 mutations are globally recognized markers for BDQ resistance and are critical for guiding diagnosis and treatment (Miotto et al., 2024). Other resistance mechanisms include those for D-cycloserine (DCS) and para-aminosalicylic acid (PAS). Genome-wide association studies suggest that mutations in fbiB and ddn may contribute to DCS resistance, though their mechanisms require further investigation (Desjardins et al., 2016). PAS resistance is linked to mutations in thyA which encodes thymidylate synthase) and folC which encodes dihydrofolate synthase (Martini et al., 2022).

The polygenic nature of Mtb drug resistance is underscored by the synergistic effects of multiple mutations across genes such as katG, inhA, and rpoB, which can enhance resistance levels and influence bacterial fitness (Gomes et al., 2021). Structural and computational analyses reveal that non-synonymous mutations in these genes induce conformational changes in enzyme targets, reducing drug binding affinity (Singh et al., 2021). For instance, mutations outside the canonical RRDR in rpoB, like P280L and D595Y, also contribute to resistance, indicating a broader mutational landscape (Zeng et al., 2021). Rapid molecular platforms such as PCR sequencing GeneXpert and nanopore sequencing now detect clinically relevant mutations directly from specimens and thus expedite the management of MDR-TB and XDR-TB (Dou et al., 2025; Ren et al., 2024). However, challenges persist due to mutation diversity, novel variants, and loss-of-function mutations that complicate genotypic predictions (Thwe et al., 2021). In summary, mutations in various genes are central to drug resistance in Mtb, altering drug-target interactions and emphasizing the need for comprehensive molecular approaches in TB control.

### 2.3 Epigenetic regulation and drug resistance

Epigenetic regulation plays a critical role in modulating gene expression in Mtb, influencing its ability to survive, evade host immunity, and develop drug resistance. DNA methylation refers to the addition of methyl groups to DNA molecules, which can alter gene transcription without changing the underlying DNA sequence (Poetsch and Plass, 2011). In Mtb, methylation has been shown to regulate numerous biological processes essential for pathogenesis, including immune evasion and drug response. It is demonstrated that Mtb can modulate both its own methylome and that of the host, affecting immune-related genes, which are pivotal in orchestrating the host defense against infection (Asaad et al., 2020). The methylation status of genes involved in drug resistance pathways can alter their expression, contributing to the pathogen’s ability to withstand antibiotic pressure. For example, genome-wide methylation analyses in streptomycin-resistant Mtb strains revealed differential methylation patterns in hundreds of genes, many of which are implicated in metabolic and regulatory pathways that may influence drug susceptibility (Wu et al., 2024).

Moreover, epigenetic modifications extend to include histone modifications and non-coding RNAs, which are increasingly recognized as key players in Mtb’s adaptation to hostile host environments and chemotherapy. Mtb infection induces host miRNA expression changes that can suppress immune responses and promote bacterial survival. The pathogen exploits these host miRNAs to modulate drug extrusion, permeability barriers, and metabolism, thereby contributing to MDR (Sukdeo et al., 2025). Furthermore, the small RNA MTS1338 is upregulated during macrophage infection and promotes Mtb resistance to acidic, nitrosative, and oxidative stresses by activating transcriptional regulators and stress chaperones, thereby facilitating adaptation within host environments (Martini et al., 2023). Additionally, Mtb itself may influence the host epigenome to silence or activate immune-related genes, effectively promoting immune escape and persistence. For instance, Mtb upregulates host sirtuin 2 (SIRT2), a NAD + -dependent histone deacetylase, which modifies histone acetylation and transcription factors such as NFκB-p65, altering macrophage activation and T cell differentiation to favor bacterial survival (Bhaskar et al., 2020).

Epigenetic regulation of immune genes critically influences host-pathogen interactions by impairing host defenses, thereby facilitating Mtb latency and antibiotic resistance. The reversible nature of these modifications presents therapeutic opportunities to restore immunity and counter resistance (Fischer et al., 2016). Furthermore, methyltransferases involved in RNA methylation, such as 16S rRNA methyltransferases, represent novel drug targets since they contribute to translation inhibition and resistance emergence (Salaikumaran et al., 2022). Deciphering the epigenetic landscape of Mtb supports the development of epigenetic therapies and host-directed strategies to augment current treatments and vaccine design (Sui et al., 2022). Overall, the epigenetic regulation in Mtb is a sophisticated survival toolkit that enables the pathogen to adapt rapidly to environmental stresses, evade immune surveillance, and develop resistance.

While epigenetic biomarkers hold promise for improving the diagnosis of active tuberculosis, their translation into public health practice depends on implementation feasibility, particularly in high-burden and low-resource settings. Widespread adoption will require affordable assay formats, compatibility with existing diagnostic infrastructure, and regulatory approval within national TB control frameworks. Although many epigenetic assays currently rely on advanced laboratory platforms, simplified PCR-based or targeted approaches may enable decentralization and integration into established molecular diagnostic networks. Importantly, epigenetic diagnostics have the potential to support the objectives of the WHO End TB Strategy, especially early and accurate case detection and improved identification of difficult-to-diagnose populations, thereby contributing to long-term TB control and elimination efforts.

### 2.4 Cell wall remodeling and metabolic reprogramming

The intrinsic drug resistance of Mtb is profoundly influenced by two interconnected adaptive strategies: dynamic cell wall remodeling and extensive metabolic reprogramming. These processes collectively enable Mtb to limit antibiotic penetration, reduce intracellular drug efficacy, and establish persistent, drug-tolerant populations. The mycobacterial cell wall is composed of peptidoglycan, arabinogalactan, mycolic acids, and an array of complex lipids, which constitutes a formidable hydrophobic barrier (Abrahams and Besra, 2021). In response to environmental stresses such as nutrient deprivation, hypoxia, or drug exposure, Mtb actively remodels this structure. This remodeling involves modifications in mycolic acid biosynthesis and trafficking, regulated by serine/threonine protein kinases including PknB, which phosphorylates transporters like MmpL3 to modulate lipid export and cell wall composition (Le et al., 2020; Devlin et al., 2024). Furthermore, enzymatic modifications, such as lipid methylation by Rv1523 methyltransferase or esterase activity of PE11 from the PE/PPE family, alter the cell wall’s physicochemical properties, directly reducing drug influx and enhancing antibiotic resistance (Ali et al., 2021; Dahiya et al., 2024). The integrity and permeability of the cell envelope are also maintained by proteins like the MmpL family transporters and VirR from the LCP family, which facilitate lipid transport and regulate the linkage between peptidoglycan and arabinogalactan (Salgueiro et al., 2024). Disruption of these components increases cell wall permeability and antibiotic susceptibility, underscoring their critical role in intrinsic resistance.

Concurrently, Mtb undergoes metabolic reprogramming to survive under these hostile conditions, facilitating a shift to a non-replicating persistent (NRP) state. This metabolic adaptation is orchestrated by regulators such as the serine/threonine protein kinase PknG, which remodels the bacterial proteome in response to hypoxia and alters nitrogen metabolism (Khan and Nandicoori, 2021; Lima et al., 2021). Central to this shift are changes in central carbon metabolism, including an increased reliance on the glyoxylate and methylcitrate cycles. These changes allow Mtb to utilize host-derived fatty acids for energy and biosynthetic purposes within niches like foamy macrophages (Laval et al., 2021). The pathogen’s energy metabolism is also reconfigured, with the cytochrome bd oxidase becoming essential for maintaining respiration under acidic stress in activated macrophages (Cai et al., 2021). This metabolic state is heterogeneous, such as higher ATP/ADP ratios in phagosomes compared to the metabolic quiescence observed in phagolysosomes (Akela and Kumar, 2021). Phenotypic heterogeneity which is driven by metabolic shifts like the trehalose catalytic shift, further promotes the emergence of drug-tolerant persisters and can facilitate the development of multidrug resistance (Lee et al., 2025).

Critically, cell wall remodeling and metabolic reprogramming are synergistic. The proteomic changes induced by metabolic stresses include alterations in enzymes responsible for synthesizing peptidoglycan, arabinogalactan, and mycolic acids, leading to pronounced cell wall restructuring that further impedes antibiotic penetration (Devlin et al., 2024). Conversely, the persistent populations formed through metabolic dormancy maintain a diverse proteome, including chaperones and oxidative stress protection enzymes, which supports survival in a state of low metabolic activity and prepares the bacteria for reactivation (Trutneva et al., 2020). The resulting drug-tolerant persister cells exhibit subtype-specific alterations in cell wall biogenesis and energy metabolism, which creates a formidable barrier to antibiotic treatment (Savijoki et al., 2021). Collectively, the interplay between a dynamically remodeled, less permeable cell wall and a reprogrammed, quiescent metabolism constitutes a central pillar of Mtb’s strategy for surviving antibiotic and immune pressures.

## 3 TB vaccine development

The BCG vaccine, developed in the early 20th century from an attenuated strain of *Mycobacterium bovis*, has been the cornerstone of TB prevention worldwide, particularly in high-burden countries. It remains essential for protecting children from severe TB, including disseminated and meningeal disease, by reducing TB-related mortality (Sadigurschi et al., 2025). Its protection stems from inducing cell-mediated immunity, Th1 responses, and antigen-specific T cells, alongside trained innate immunity (Faustman, 2020). Mucosal administration can also promote lung-resident memory T cells, vital for local defense (Blake et al., 2025). However, BCG fails to consistently prevent adult pulmonary TB, with efficacy declining over time. This limitation arises from several factors, including intradermal administration weakens mucosal immunity in the lungs (Kurtz et al., 2023), host genetic diversity and bacterial variability affect immune responses (Azad et al., 2020), and Mtb’s immune evasion strategies subvert vaccine-induced protection (Zhang W. et al., 2025). Additionally, the variable genetic makeup of BCG vaccine strains themselves, with differences in virulence and immunogenicity, contributes to inconsistent efficacy across regions (Bernatowska et al., 2022). The absence of clear immune correlates further complicates vaccine improvement (Lai et al., 2023). Thus, while BCG is invaluable in pediatric TB control, its inadequate performance against adult pulmonary TB underscores the necessity for more effective vaccination approaches.

The development of novel TB vaccines has become a critical priority due to the limited efficacy of the BCG vaccine. Key candidates and platforms are summarized in Table 1. Among the promising new candidates are protein subunit vaccines such as M72/AS01E, which have demonstrated encouraging clinical trial results. M72/AS01E has shown significant efficacy in preventing active TB disease in adults with latent Mtb infection, eliciting robust antigen-specific Th1 immune responses characterized by IFN-γ production (Scriba et al., 2020). In parallel, multi-epitope vaccines and mRNA-based vaccine design strategies are gaining attention for their capacity to induce broad and potent immune responses. Multi-epitope vaccines incorporate multiple T- and B-cell epitopes derived from various Mtb antigens, enhancing immunogenicity and coverage across diverse populations (Pillay et al., 2024). A computational approach have facilitated the identification and validation of such epitopes, enabling the construction of fusion proteins or chimeric antigens that stimulate both humoral and cellular immunity (Ghandadi, 2022). Similarly, mRNA vaccine platforms offer rapid development and the ability to encode multiple antigens, which can be tailored to induce specific immune profiles. In silico design of multi-epitope mRNA vaccines directed against essential Mtb proteins has revealed robust antigenicity and stability alongside predicted high immunogenicity while molecular docking indicated strong TLR4 engagement (Zhu et al., 2024). DNA vaccines and nanomaterial-based delivery systems also represent innovative approaches to TB vaccination. DNA vaccines encoding Mtb antigens and latency-associated proteins have been shown to induce antigen-specific T-cell responses that contribute to bacterial clearance in animal models (Karanika et al., 2024; Mobed, 2020). Liposome-encapsulated subunit vaccines have enhanced strong Th1-type responses essential for TB control in murine model (Gurmessa et al., 2025). Extracellular vesicles derived from Mtb-infected macrophages carry immunodominant antigens and possess intrinsic adjuvant properties, making them attractive as cell-free vaccine candidates that stimulate both innate and adaptive immunity (Ji et al., 2024a; Ji et al., 2024b). Furthermore, recombinant viral vectors configured as prime-boost regimens amplify BCG-primed immunity and have elicited robust immunogenicity and protection in preclinical model (Shin et al., 2025). Collectively, these novel vaccine candidates and platforms represent a multifaceted approach to TB vaccine development.

**TABLE 1:** Promising platforms and candidates in the current TB vaccine development.

Strategy	Candidates	Stage	Key features	Challenges
Protein subunit	M72/AS01E	Clinical	Significant efficacy in latently infected adults; induces robust Th1/IFN-γ response	Antigenic variation may affect efficacy
Viral vector	Recombinant viral vectors	Clinical	Amplifies BCG-primed immunity; elicits robust immunogenicity	Pre-existing immunity to the vector may reduce effectiveness
mRNA-based	Multi-epitope mRNA vaccines	*In silico*	Rapid development; encodes multiple antigens; high predicted immunogenicity and TLR engagement	Requires extensive *in vitro* and *in vivo* validation
DNA vaccine	DNA encoding Mtb antigens	Preclinical	Induces antigen-specific T-cell responses; contributes to bacterial clearance in models	Need for improved delivery systems to enhance immunogenicity in humans
Nanomaterial delivery	Liposome-encapsulated subunits; extracellular vesicles	Preclinical	Enhanced Th1 responses; intrinsic adjuvant properties; targeted delivery	Scalable manufacturing, stability, and quality control
Multi-epitope	S7D5L4; MP3RT; chimeric constructs	*In silico*	Targets multiple epitopes for broad immunity; combines B-cell, CTL, and HTL responses	Transition from promising computational predictions to clinical efficacy
Computational design	Immunoinformatics, molecular docking, MD simulations	*In silico*	Revolutionizes epitope identification and vaccine design; predicts stability and binding	Results must be validated experimentally; high computational resource demands

The integration of immunoinformatics and computational simulation has revolutionized vaccine design. Multiple studies have utilized bioinformatics tools to identify epitopes from various Mtb proteins, such as EIS (Rv2416c), MPT83, MPT51, and secreted exosome proteins, which are critical in pathogenesis and immune evasion (Logesh et al., 2022; Loureiro et al., 2025; Sharma et al., 2021). The predicted epitopes are then assembled into multi-epitope vaccine constructs, often incorporating immunostimulatory adjuvants such as TLR agonists to enhance immunogenicity (Khan et al., 2023). Structural modeling and refinement of the vaccine constructs are crucial steps that follow epitope prediction. Molecular docking studies simulate the interaction between vaccine constructs and immune receptors. For instance, docking of a chimeric vaccine based on EIS protein with TLR4 demonstrated stable binding, which was further supported by molecular dynamics (MD) simulations confirming the stability of the vaccine-receptor complex over time (Logesh et al., 2022). Similarly, molecular docking and MD simulations have been employed to validate the binding stability of multi-epitope vaccines with TLR2, TLR3, and TLR4, underscoring their immunostimulatory potential (Mubarak et al., 2024). MD simulations provide a dynamic perspective on the stability and conformational flexibility of vaccine-receptor complexes under physiological conditions. MD simulations of a multi-epitope vaccine construct targeting drug-resistant Mtb strains revealed stable interactions with TLR4, supporting its candidacy for further experimental validation. Additionally, MD simulations have been instrumental in confirming vaccine stability in other infectious diseases (Khan et al., 2021), highlighting the broad applicability of this approach. Despite the promising *in silico* results, the necessity of *in vitro* and *in vivo* validation remains paramount. The multi-epitope vaccine S7D5L4, designed against co-infection of Mtb and SARS-CoV-2, demonstrated excellent antigenicity and immunogenicity *in silico*, but the authors highlighted the need for further experimental validation to confirm its protective efficacy (Peng et al., 2023). Similarly, the MP3RT vaccine showed concordance between immunoinformatics predictions and animal immune responses, which emphasizes the value of combined computational and experimental approaches (Cheng et al., 2022).

The development of effective vaccines against Mtb faces formidable challenges primarily due to the complex biology of the pathogen and the intricacies of the host immune response. One major obstacle is the antigenic diversity and genetic variability of Mtb strains, which can significantly impact vaccine efficacy. The M72/AS01E candidate exemplifies this challenge, as genetic polymorphisms in its PPE18 antigen alter epitope binding and TLR2 interactions, potentially inducing non-protective Th2 immunity (Theorupun et al., 2025). Thus, vaccine design must account for such antigenic variation to ensure broad protection across circulating Mtb lineages. Another critical challenge is the lack of well-defined immunological correlates or biomarkers of protection. The incomplete understanding of protective immune mechanisms against Mtb significantly hinders vaccine development. It is exemplified by the Ag85B antigen, whose immunogenicity has been found to compromise BCG vaccine efficacy by activating regulatory T cell responses and macrophage exhaustion pathways. These include the expansion of IFN-γ/IL-10-dual-producing regulatory T cells and upregulation of PD-L1, ultimately promoting bacterial survival (Piccaro et al., 2024). The absence of such validated immune markers further obstructs the establishment of clinical trial endpoints, substantially prolonging vaccine development timelines. The design of vaccines that are effective across different human populations adds another layer of complexity. Diverse host factors such as genetic background, age, co-infections, and nutritional status influence vaccine responsiveness (Jiang et al., 2025). HIV-infected individuals exhibit altered immune landscapes that may reduce vaccine efficacy (Miner et al., 2022). Additionally, sex differences have been observed in vaccine-induced immunity, with a study showing that recombinant BCG derivatives confer improved protection in male mice compared to BCG alone (Harikumar Parvathy et al., 2025). Challenges also arise in vaccine formulation and delivery. The incorporation of multiple antigens to broaden immune coverage shows promise but requires careful evaluation of immunogenicity and safety profiles (Ding et al., 2025). Additionally, novel adjuvants and delivery routes aim to elicit localized immunity at the primary site of infection, which must overcome mucosal tolerance and immune activation (Stylianou and Satti, 2024). The lack of standardized and predictive animal models and controlled human infection models further complicates preclinical and clinical evaluation.

The clinical development and global implementation of TB vaccines face multifaceted challenges. Ethical complexities arise in trial design, particularly concerning vulnerable groups like incarcerated individuals and people living with HIV, requiring stringent safeguards and specialized protocols (Andrews et al., 2025). Operational barriers, including participant recruitment difficulties as seen in the MVA85A trial, further complicate clinical evaluation (Riste et al., 2021). Socioeconomic hurdles encompass high production costs threatening affordability in high-burden regions and inadequate distribution infrastructure (Hoseinpour et al., 2024). Effective deployment requires integrating vaccination into local health systems, targeting key groups, and enhancing international collaboration to ensure equitable access (Weerasuriya et al., 2020). In conclusion, TB vaccine development is navigating a landscape marked by both innovation and challenges. Emerging technological platforms and computational tools offer renewed promise while the high benchmark set by BCG makes further breakthroughs considerably difficult. Future progress requires addressing both scientific hurdles and implementation barriers. Final success will depend on synergistic collaboration between scientific innovation and public health practice.

## 4 Translational challenges in TB vaccine development

Despite advances in immunoinformatics and next-generation vaccine platforms, the translation of TB vaccine candidates into clinical success remains challenging (Ortega-Tirado et al., 2020). A major limitation is the poor predictive value of commonly used animal models, which often fail to accurately reflect human immune responses and disease progression (Ortega-Tirado et al., 2020). In addition, validated immune correlates of protection against TB are still lacking, complicating both rational vaccine design and the interpretation of clinical trial outcomes (Cha et al., 2023). Historically, a substantial number of TB vaccine candidates that showed promising preclinical or early-phase clinical results have failed to demonstrate efficacy in late-stage trials, underscoring the high attrition rate in this field (Cha et al., 2023). These challenges highlight the need for cautious interpretation of emerging vaccine technologies and emphasize the importance of well-designed clinical studies, improved biomarkers of protection, and iterative validation in human populations.

## 5 Development of new anti-TB drugs

The rise of MDR and extensively XDR strains of Mtb has made the development of new anti-TB drugs an urgent priority. The research landscape now spans a broadening array of strategies. As summarized in Table 2, these approaches span from preclinical discovery to clinical application, each with distinct advantages and challenges. This multi-pronged effort is crucial for building a robust pipeline to combat drug-resistant TB. Advances in proteomics and genomics have enabled the identification of novel drug targets, such as membrane proteins and enzymes essential for bacterial survival. For instance, the mycobacterial membrane protein large 4 (MmpL4), involved in lipid transport and cell wall biosynthesis, has been explored through computational approaches like structure-based pharmacophore modeling and molecular docking (Woodworth et al., 2021). These methods have identified potential inhibitors, including SQ109 derivatives, which show promising binding affinities and stability in molecular dynamics simulations (Woodworth et al., 2021). Additionally, anti-virulence therapy has emerged as a strategy to combat resistance by targeting bacterial virulence factors rather than killing the pathogen directly. This approach reduces selective pressure for resistance development and has identified twelve virulence factors as potential drug targets, some of which are suitable for intracellular action or vaccine development (Bhattacharya et al., 2020). Omics technologies have further revealed molecular adaptations of Mtb under drug pressure, leading to targets like decaprenyl-phosphoryl-β-D-ribose 2′-epimerase (DprE1), which is a key enzyme in cell wall biosynthesis (Liu et al., 2022). Inhibitors designed to interact with flexible regions of DprE1 have demonstrated enhanced potency against resistant strains (El et al., 2024). Another innovative approach involves proteolysis targeting chimeras adapted for mycobacteria (Bac-PROTACs), which recruit bacterial proteolytic machinery to degrade essential proteins like ClpC1, effectively killing drug-resistant Mtb strains (Junk et al., 2024).

**TABLE 2:** Current strategies for anti-TB drug discovery and development.

Research Direction	Specific strategy	Drugs/Candidates	Stage	Key features	Challenges
Targeting resistance mechanisms	MmpL3/MmpL4 inhibitors	SQ109 and its derivatives	Preclinical	Novel mechanism overcoming efflux pump-mediated resistance	Require validation of *in vivo* efficacy and safety
	Anti-virulence therapy	Inhibitors targeting 12 essential hypothetical proteins	Preclinical	Reduced selective pressure, lower resistance development	Complex functional validation of virulence factors
	DprE1 inhibitors	Benzothiazinone derivatives	Clinical	Potent inhibition of cell wall synthesis, broad-spectrum activity against resistant strains	Potential resistance via mutations
	Targeted protein degradation	BacPROTACs	Preclinical	Complete elimination of target proteins, overcoming traditional resistance	Delivery system optimization and selectivity concerns
Novel therapeutic agents and combination	ATP synthase inhibitors	Bedaquiline	Approved	Novel mechanism, effective against dormant bacilli	QT interval prolongation and other cardiac toxicities
	Mycolic acid synthesis inhibitors	Delamanid	Approved	Effective under hypoxic conditions	Need for pediatric formulations
	Short-course regimens	BPaL regimen	Clinical	6-month treatment duration, high cure rates	Monitoring for adverse effects including myelosuppression
	Nano-drug delivery systems	Various nanocarriers	Preclinical	Enhanced bioavailability, targeted delivery	Scalable manufacturing and quality control
CADD	Molecular docking virtual screening	Inhibitors targeting EF-G, DprE1, Pks13	-	High-efficiency lead compound screening	False positive rates require control
	Molecular dynamics simulations	Optimized compounds for Rv1250, MurB targets	-	Reveals dynamic binding mechanisms, guides optimization	High computational resource demands
	AI and QSAR modeling	Machine learning prediction models	-	Multi-parameter optimization, improved success rates	Model interpretability needs enhancement
HDT	Autophagy inducers	SMIP-030/SMIP-031	Preclinical	Enhances host clearance capacity, reduced resistance development	Difficulties in immune balance regulation
	Immune checkpoint modulation	CD84 blockers	Preclinical	Restores T-cell function	Autoimmunity risks
	Metabolic reprogramming	Vitamin D analogs	Clinical	Known safety profile, dual mechanisms of action	Significant individual efficacy variations
	Cellular therapies	MDSCs depletion strategies	Preclinical	Breaks immune tolerance	Technical complexity

In terms of novel therapeutics, drugs such as bedaquiline and delamanid have been incorporated into regimens for drug-resistant TB. Bedaquiline targets the mycobacterial ATP synthase to inhibit oxidative phosphorylation and lead to bacterial death (Harikishore et al., 2023). This mechanism is distinct from traditional first-line drugs, which target cell wall synthesis or nucleic acid metabolism, thereby making bedaquiline effective against strains resistant to conventional therapy (Harikishore et al., 2023). On the other hand, delamanid inhibits mycolic acid synthesis through the inhibition of enzymes involved in the biosynthesis pathway. Its bactericidal activity is particularly notable under hypoxic conditions, which are common in granulomatous lesions (Sachan et al., 2023). The World Health Organization has updated guidelines to include these agents in combination therapies to mitigate resistance risks (Lange, 1946). Besides, the combination of bedaquiline, pretomanid, and linezolid led to a favorable outcome at 6 months after the end of therapy in a high percentage of patients with highly drug-resistant forms of tuberculosis (Conradie et al., 2020). Synergistic interactions in these combinations enhance bactericidal activity against persister populations (Conradie et al., 2020). Furthermore, computational modeling supports the use of optimized combination therapies to maximize efficacy while minimizing toxicity and resistance development (Wani et al., 2025). Adjunctive therapies, such as nanocarrier-based drug delivery systems, improve bioavailability and targeted delivery, further enhancing treatment outcomes (Helikumi et al., 2025).

Computer-aided drug design (CADD) and molecular simulation play crucial roles in anti-tubercular drug discovery. Molecular docking allows virtual screening of compound libraries to identify inhibitors targeting essential Mtb proteins, such as elongation factor G (EF-G) and DprE1 (Altharawi et al., 2023). For example, benzothiazinone analogs identified through docking show irreversible inhibition of DprE1 (Altharawi et al., 2023). Molecular dynamics simulations complement this by providing insights into the stability and flexibility of protein-ligand complexes under physiological conditions. MD analyses revealed that mutations in HIV-1 protease and HCV NS5B polymerase alter binding site conformations and reduce inhibitor affinity, providing mechanistic understanding of resistance (Kobayashi et al., 2021). In the context of Mtb, MD simulations have been instrumental in characterizing the binding dynamics of natural compounds and synthetic derivative (Ibrahim et al., 2024). Integration with techniques like quantitative structure-activity relationship (QSAR) modeling and machine learning enhances predictive power for drug-likeness and pharmacokinetic profiles (Adeniji, 2021). These approaches have facilitated the design of multi-targeting agents and the evaluation of drug-likeness, toxicity, and pharmacokinetic profiles early in the drug development pipeline (Amandy et al., 2024). Additionally, fragment-based drug discovery (FBDD) combined with computational methods has been recognized as a promising strategy for identifying novel scaffolds against Mtb targets (Togre et al., 2022).

Host-directed therapeutic (HDT) strategies focus on modulating the host immune system to improve TB outcomes. Immunomodulatory agents, such as repurposed drugs and small molecules, target pathways like autophagy and inflammatory signaling. SMIP-030 and its derivative SMIP-031 inhibit host phosphatase PPM1A to induce autophagic clearance of Mtb in macrophages (Yu et al., 2024). Nanoparticle-based delivery systems enhance autophagy induction by improving drug stability and targeting (Maphasa et al., 2020). Depletion of myeloid-derived suppressor cells (MDSCs) using a diphtheria toxin fusion protein targeting IL-4 receptor-positive cells enhances T cell-mediated immunity and reduces bacterial burden in TB models (Parveen et al., 2021). Additionally, immune checkpoint molecules such as CD84 have been identified as suppressors of T and B cell activation during Mtb infection; blocking such inhibitory receptors may reinvigorate adaptive immunity and improve bacterial clearance (Zheng et al., 2022). Combination of immunomodulators with standard antibiotics has shown synergistic effects by promoting antimycobacterial immune responses (Dwivedi et al., 2023). Additionally, vitamin D analogs and metabolic reprogramming of macrophages offer novel avenues for host-directed intervention (Reddy et al., 2024).

The management of drug-resistant TB in children presents unique challenges due to the paucibacillary nature of the disease and difficulties in diagnosis. Molecular diagnostics have improved detection of Mtb and rifampicin resistance though sensitivities vary by specimen type in pediatric samples (Kay et al., 2022). Complementary tests like Genotype MTBDRplus are needed to detect broader resistance mutations (Khurana et al., 2021). New drugs like bedaquiline and delamanid enable shorter and all-oral regimens for children, with studies showing favorable outcomes and high sputum culture conversion rates (Kalawadia et al., 2024). However, challenges remain in dosing optimization, safety monitoring, and the development of child-friendly formulations (Buonsenso et al., 2023). Preventive therapies such as tuberculosis preventive therapy (TPT) with levofloxacin or delamanid, have shown promise in reducing transmission risks (Apolisi et al., 2023). Besides, Psychosocial and nutritional support are also essential components of comprehensive care for pediatric patients (Dharmapalan and Mane, 2023). Overall, progress in anti-TB drug development involves a multipronged approach: leveraging omics data for target identification, employing computational tools for drug optimization, integrating novel drugs into combination therapies, and adopting host-directed strategies. Continued innovation and clinical validation are crucial to address the global burden of drug-resistant TB.

## 6 Limitations

This review has several limitations. First, it is a narrative review, and therefore the selection and interpretation of the literature may be subject to author perspective rather than systematic inclusion criteria. Second, much of the evidence discussed is derived from preclinical studies and *in silico* analyses, which provide important mechanistic insights but may not fully predict clinical outcomes in humans. Third, drug resistance mechanisms in *Mycobacterium tuberculosis* are rapidly evolving, and newly emerging mutations or adaptive pathways may not yet be captured in the current literature. In addition, for many vaccine candidates, long-term clinical efficacy data remain limited, particularly regarding durable protection against adult pulmonary TB and drug-resistant strains.

Computational approaches, including molecular docking and molecular dynamics simulations, provide valuable structural and mechanistic insights into molecular interactions and have an important role in hypothesis generation. However, these methods are inherently limited by model assumptions, force-field accuracy, and the quality of input structures, and therefore do not necessarily predict biological or clinical efficacy. As such, findings derived from *in silico* analyses should be interpreted as exploratory and hypothesis-generating rather than as evidence of direct clinical applicability. Experimental validation using biochemical, cellular, and *in vivo* models remains essential to confirm the functional relevance of computational predictions.

These limitations highlight the need for continued clinical validation, longitudinal studies, and integrated surveillance efforts to refine future therapeutic and vaccine strategies.

## 7 Conclusion

TB remains a formidable global health challenge, primarily due to the remarkable capacity of Mtb to develop resistance to antimicrobial agents and the limited efficacy of the century-old BCG vaccine. This review has synthesized the complex landscape of Mtb drug resistance, which extends far beyond simple genetic mutations. It encompasses a sophisticated array of mechanisms including epigenetic regulation, extensive metabolic reprogramming, and dynamic cell wall remodeling. These adaptations are tightly interwoven with immune-evasion tactics that collectively erect a formidable barrier to effective therapy. The emergence of MDR and XDR strains underscores the urgent need for therapeutic strategies that target not only the bacterium but also the host-pathogen interface. BCG’s limited and heterogeneous efficacy against adult pulmonary TB, the most transmissible form of the disease, exposes a major void in current TB prevention strategies. While BCG is invaluable for protecting children from severe forms of TB, its inability to consistently prevent pulmonary disease in adults has spurred intensive research into next-generation vaccines. The present pipeline comprises protein subunits, viral vectors and next-generation nucleic acid constructs each offering distinct immunological advantages. The integration of immunoinformatics and computational biology has revolutionized vaccine design, enabling the rational construction of multi-epitope vaccines that elicit broad cellular and humoral immunity. Before costly and time-consuming experimental studies, approaches *in silico* which are validated by molecular docking and dynamics simulations allow for the prediction of stable vaccine-receptor interactions and robust immunogenicity. Concurrently, the development of new anti-TB drugs has become an imperative priority. The advent of bedaquiline and delamanid, which target novel bacterial pathways, represents a significant advance in managing drug-resistant TB. Therapeutic strategy is shifting towards optimized combination regimens exemplified by BPaL to raise efficacy and eradicate persister *bacillus*. CADD and molecular simulations are indispensable tools in this endeavor, accelerating the identification and optimization of inhibitors against essential Mtb targets. Furthermore, HDT offer a complementary approach by modulating the host immune response to enhance bacterial clearance and mitigate pathological inflammation. This paradigm recognizes that overcoming TB requires a comprehensive attack on both the pathogen and the host environment it manipulates.

Looking forward, the future of TB control needs innovation and integration. The design of multi-target and multi-epitope vaccines aims to overcome antigenic variability and induce comprehensive, durable immunity. Novel platform technologies, particularly mRNA vaccines delivered via lipid nanoparticles, hold immense promise due to their rapid development cycle and potent capacity to induce T-cell responses. Nanotechnology delivery systems such as self-assembling peptides and microneedle patches enhance vaccine stability and immunogenicity while enabling mucosal administration. The concept of personalized medicine and precision vaccinology is also emerging, where interventions could be tailored based on individual host genetics, immune status, and the infecting Mtb strain. This approach could maximize efficacy across diverse global populations. Ultimately, translating these scientific advances into public health impact demands robust global collaboration and sustained policy support. Strengthening international partnerships is crucial for sharing genomic surveillance data, which tracks the evolution and spread of drug-resistant strains. Advanced molecular diagnostics such as whole-genome sequencing and droplet digital PCR empower continuous surveillance systems to monitor resistance patterns and vaccine effectiveness in real time. Such systems form the backbone of rapid response mechanisms to contain outbreaks and inform treatment guidelines. Besides, it is desirable that TB be given higher visibility in future policy revisions while steps are taken to broaden equitable access to new tools and to create an environment that nurtures research and development. In summary, the fight against TB is at a pivotal juncture. The challenges posed by drug resistance and an imperfect vaccine are significant, but they are matched by unprecedented opportunities arising from scientific and technological progress. A successful strategy will be inherently multidisciplinary, synergizing advances in microbiology, immunology, computational biology, and clinical medicine. Ending the TB epidemic becomes an increasingly attainable reality by integrating novel vaccines, smarter therapeutics, and precision public health approaches within a global framework.
